# Hypoxic Ischemic Encephalopathy: Hearing Impairment and Related Risk Factors

**DOI:** 10.3390/jcm15093180

**Published:** 2026-04-22

**Authors:** Francesca Serrao, Simonetta Frezza, Guido Conti, Simona Fattore, Mirta Corsello, Alessadra Lio, Chiara Di Sipio Morgia, Chiara Concilio, Angelo Tizio, Tommaso Verdolotti, Simona Gaudino, Simonetta Costa, Giovanni Vento

**Affiliations:** 1 Neonatal Unit, Department of Woman and Child Health and Public Health, Fondazione Policlinico Universitario Agostino Gemelli IRCCS, Catholic University of Sacred Heart, Largo Agostino Gemelli 8, 00168 Rome, Italy; 2 Clinic of Otorhinolaryngology—Audiology Service, Fondazione Policlinico Universitario Agostino Gemelli IRCCS, Catholic University of Sacred Heart, Largo Agostino Gemelli 8, 00168 Rome, Italy; 3 Radiology and Neuroradiology Department, Fondazione Policlinico Universitario Agostino Gemelli IRCCS, Catholic University of Sacred Heart, Largo Agostino Gemelli 8, 00168 Rome, Italy

**Keywords:** hearing loss, hypoxic–ischemic-encephalopathy, therapeutic hypothermia

## Abstract

**Objectives:** The purpose of this study was to compare the incidence of hearing loss at three months of age in a cohort of newborns with hypoxic-ischaemic encephalopathy (HIE) treated with therapeutic hypothermia (TH) with that reported in the literature. We also evaluated potential risk factors associated with audiological impairment and changes in hearing threshold during follow-up. **Methods:** This retrospective observational cohort study was conducted at the Neonatal Intensive Care Unit of the Fondazione Policlinico Universitario A. Gemelli, IRCCS in Rome, Italy, between January 2017 and December 2023. Infants underwent audiological screening and a full diagnostic evaluation at three months of age and were followed during the first year of life. **Results:** A total of 149 infants were enrolled, and hearing loss was identified in six (4.0%) at three months of age. Two of these six infants showed an improvement in their hearing threshold, resulting in a prevalence of permanent bilateral sensorineural hearing loss (SNHL) of four out of 149 infants (2.7%), with no cases of late-onset hearing loss detected. Gestational age was identified as an independent protective factor against SNHL (OR 0.49; 95% CI 0.22–0.91). **Conclusions:** The audiological screening program demonstrates effectiveness in early intervention for diagnosing and treating hearing loss. Infants with HIE are at high risk for hearing disorders and require increased attention in neonatological and audiological management. Management should be individualized based on specific risk factors. The association between gestational age and susceptibility to cochlear damage should be confirmed by further studies.

## 1. Introduction

Hypoxic-ischemic encephalopathy (HIE) is a severe neurological condition affecting 1–8 per 1000 term and near-term births in high-income countries, and is a significant cause of neonatal death and long-term neurodevelopmental sequelae [1]. The brain injury associated with HIE is caused by perinatal oxygen deprivation and reduced cerebral blood flow. Therapeutic hypothermia (TH) has been shown to improve outcomes in these newborns by reducing the risk of death and disability. TH is now the standard of care for all infants who meet the criteria for moderate to severe HIE in high-income countries [2,3]. However, the prevalence of hearing loss (HL) in this population remains high, although it varies across studies [4,5,6,7,8]. The variation in prevalence is likely due to differences in testing methods, timing of definitive diagnosis, and clinical management during the first months of life. Oxygen deprivation from perinatal asphyxia can damage the hair cells of the inner ear, the auditory nerve, or the brain structures that process sound. Infants with perinatal asphyxia often require intensive care and may have comorbidities or receive ototoxic medications, which can increase the risk of hearing damage [9]. Investigating possible predisposing risk factors for HL in these infants could provide guidance for improving clinical practice and help plan a more accurate audiological follow-up.

The aim of this study was to compare the incidence of hearing impairment in our cohort of newborns with HIE treated with TH to that reported in the literature, identify risk factors for HL, and analyze changes in hearing threshold during audiological follow-up.

## 2. Methods

### 2.1. Study Design, Setting and Population

This retrospective observational cohort study was conducted at the Neonatal Intensive Care Unit (NICU) of the Fondazione Policlinico Universitario A. Gemelli, IRCCS, in Rome, Italy, from 1 January 2017 to 31 December 2023, in compliance with STROBE guidelines for reporting observational studies [10].

All infants with HIE admitted to our NICU and treated with TH were included. Patients with congenital infections or congenital abnormalities were excluded, as were cases in which parents did not give consent for anonymous data use, those who died before audiological screening, and those with missing data necessary for the study.

### 2.2. Ethics

The study was conducted in accordance with the Declaration of Helsinki and does not contain any personal information that could identify the patient. All data analyzed were collected as part of routine diagnosis and treatment, and patient medical care was not provided for research purposes but was part of standard clinical procedure. The study protocol was approved by the Institutional Review Board (Protocol 12692/22, ID 4867). Written informed consent was obtained from the parents.

### 2.3. Primary and Secondary Outcomes

The primary outcome of the study was to compare the incidence of HL at three months of age in our cohort of newborns with HIE to that reported in the literature. Secondary outcomes were to identify potential risk factors for HL and to evaluate changes in hearing threshold during audiological follow-up.

### 2.4. Hearing Screening and Diagnostic Methods

All enrolled infants underwent the NICU dual-stage hearing screening protocol. The audiological evaluation included medical history, otoscopy, transient evoked otoacoustic emissions (TEOAEs), diagnostic (laboratory) ABR recording, and, if necessary, impedance (tympanometry) and stapedial reflex testing.

The first TEOAE recordings were performed before discharge using the Madsen Accuscreen^®^ (GN Otometrics A/S, Taastrup, Denmark), which evaluates the patient’s TEOAEs through noise-weighted averaging and counting significant signal peaks. The stimulus was a non-linear click sequence at a rate of 60 Hz, delivered through the probe at a sound pressure level of 70–84 dB SPL, with self-calibration based on ear canal volume.

The first ABR recording was performed within 3 months of age, in a soundproof and electrically shielded room. Natural sleep or quietness during the recording session was monitored in each infant by visual inspection and EEG signal. Both ears were tested sequentially. Stimuli consisted of 0.1 ms clicks presented with alternating polarity through earphones (TDH-49P) at a rate of 21.1/s. The recording system was an ICS Chartr EP equipped with an ICS Chartr PA-800 preamplifier (GN Otometrics A/S, Taastrup, Denmark). Surface electrodes were placed at the vertex (+), ipsilateral ear lobe (−), and contralateral ear lobe (ground), with inter-electrode impedance kept below 5 kΩ. The signal was amplified (100 k) and filtered (50–3000 Hz). Each recording was obtained by averaging 500–1500 single epochs within a 15 ms time window and was replicated at least twice, mainly at the electrophysiological threshold level. This threshold was determined as the lowest intensity level at which a response could be identified and replicated by the presence of the V wave. Starting from a 60 dBnHL level, the threshold was assessed using a “20 dB down–10/5 dB up” procedure. At the end of the session, a recording at 80 dBnHL was usually performed for each side to allow better evaluation of the morphology and latency of the ABR response. ABR recording and analysis were carried out by an audiology technician under the supervision of an expert physician. When required (mainly in cases of abnormal electrophysiological threshold), audiological assessment was supplemented by tympanometry and stapedial reflex testing, performed using a Tympstar Granson Saddler^®^ (Grason-Stadler, Inc., Eden Prairie, MN, USA) with a 226 Hz or 660 Hz probe tone (depending on the child’s age) delivered at 85 dB SPL ± 1.5 dB.

All infants were referred for follow-up to obtain a definitive audiological diagnosis and begin treatment for hearing loss within 6 months of age. Audiological follow-up continued until 12 months of age, with assessments every 3 to 6 months depending on individual clinical condition, to identify any changes in hearing threshold.

The hearing threshold (dB HL) was estimated by subtracting 10 dB from the electrophysiological threshold (dB nHL) [11,12]. HL was defined as mild (20–40 dB), moderate (41–70 dB), severe (71–90 dB), and profound (>90 dB) [13].

### 2.5. Data Collection and Definitions

Maternal data included mode of delivery and the presence of pathologies that could cause intrapartum asphyxia, such as metabolic conditions (gestational and pregestational diabetes), cardiovascular conditions (pregestational hypertension and preeclampsia), and infectious diseases (chorioamnionitis or TORCH complex infections). Other maternal-fetal risk factors identified were shoulder dystocia and perinatal asphyxia sentinel events. Fetal bradycardia, pathological cardiotocography, meconium-stained amniotic fluid, and placental abruption were considered sentinel events of asphyxia.

Neonatal data included gender, gestational age (GA) determined by the best obstetric estimate based on the first day of the last menstrual period, prenatal ultrasound, and postnatal physical examination, and birth weight (BW). Abnormal fetal growth resulting in small for gestational age (SGA) or large for gestational age (LGA) BW was also recorded. Acid-base parameters of umbilical cord blood and Apgar scores at 1, 5, and 10 min were documented. SGA infants were defined as those with BW less than the 10th percentile, and LGA infants were defined as those with BW greater than the 90th percentile, based on Intergrowth-21 charts [14].

The occurrence of early onset sepsis, respiratory distress syndrome (RDS), persistent pulmonary hypertension, pulmonary air leak, acute kidney injury (AKI), hypoglycemia, hyperglycemia requiring insulin, hypotension requiring inotropic support, anemia requiring red blood cell transfusion, thrombocytopenia requiring platelet transfusion, and coagulopathy requiring fresh frozen plasma transfusion was recorded. Proven early onset sepsis was defined as sepsis occurring before 72 h of life and confirmed by a positive blood culture. RDS was defined based on clinical assessment of work of breathing and inspired oxygen requirement, along with radiographic or ultrasound imaging findings compatible with RDS [15]. AKI was defined according to KDIGO AKI Guidelines [16].

The need for mechanical ventilation, diuretic therapy with furosemide, duration of oxygen therapy and parenteral nutrition, number of antibiotic cycles per infant, and length of hospital stay were also recorded.

TH was initiated within 6 h of life in infants with GA ≥ 35 weeks and birth weight ≥ 1800 g if both of the following criteria were met: (1) intrapartum hypoxia, defined by at least one of the following: (A) Apgar score ≤ 5 at 10 min, (B) persistent need for resuscitation at 10 min, or (C) blood gas acidosis, characterized by either pH ≤ 7.0 or base excess ≤ −12 mmol/L in the first hour of life; (2) moderate to severe encephalopathy, evaluated between 30 and 60 min after birth [17]. Neurological involvement was confirmed by amplitude-integrated electroencephalogram (aEEG). Outborn neonates began passive cooling at the transferring center, avoiding heating and targeting a body temperature of 35 °C. The target rectal temperature was 33.5 °C (±0.5 °C), achieved with the CritiCool (Belmont Medical Technologies, Billerica, MA, USA) system and maintained through servo-controlled temperature monitoring and regulation. Treatment continued for 72 h, followed by slow rewarming with gradual increases of no more than 0.5 °C per hour until normothermia was achieved. During TH, patients received routine clinical care, including monitoring of vital signs and surveillance for organ dysfunction. To provide intravenous therapies, a central venous catheter was inserted; a peripheral venous line and a central arterial catheter were placed when necessary. Clinical, instrumental, and blood parameters were checked to identify known consequences of asphyxia or secondary effects of the treatment. In particular, heart rate, respiratory rate, urinary output, and systemic blood pressure were monitored. Blood glucose levels, electrolytes, coagulation tests, and blood cell counts were assessed and corrected if necessary. Respiratory management was individualized according to the patient’s general and neurological conditions, taking into account possible overlapping pulmonary diseases.

All newborns received personalized parenteral nutrition, starting with a total fluid supply of 50–55 mL/kg, considering the possible presence of dyselectrolytemia, acute kidney injury, or syndrome of inappropriate antidiuretic hormone secretion. Symptomatic seizures were treated with phenobarbital as the first-line drug; if ineffective, midazolam was used. Broad-spectrum antibiotic prophylaxis was routinely administered with ampicillin (50 mg/kg/day) and amikacin (15 mg/kg every 36 h) [18,19] until cultures were negative. Hypotension was evaluated using both invasive and noninvasive measurements. The need for inotropic support was also assessed by echocardiography. Pain assessment and management included serial evaluation with the NPASS score, minimizing the number of painful stimuli, and sedation with an opioid administered continuously via a dedicated line at the minimum necessary dose (starting at 0.075 µg/kg/min of remifentanil), with additional boluses as needed (e.g., fentanyl 2 µg/kg). Early enteral feeding via nasogastric tube for trophic purposes was provided to all babies without abdominal pathologies or inotropic support.

The severity of neonatal encephalopathy was assessed using the modified Sarnat score [20] within one hour of birth, either at the transferring center or upon admission to the NICU. Neurological function was monitored by continuous aEEG within the first 6 h of life and throughout the entire 72 h of therapeutic hypothermia. After rewarming, an electroencephalogram was performed and reviewed by an experienced pediatric neurologist, and magnetic resonance imaging (MRI) was conducted between days 7 and 10 after birth.

Neuroimaging results were evaluated according to Rutherford et al. [21,22]. Moderate or severe lesions in the basal ganglia and thalamus (BGT), an abnormal posterior limb of the internal capsule (PLIC), or severe white matter (WM) lesions were considered predictive of an abnormal neurodevelopmental outcome [23].

All data were collected retrospectively from electronic records by a team of neonatologists.

### 2.6. Sample Sizes and Statistical Analysis

The sample size was calculated based on an SNHL incidence of 3% in neonates with risk factors and an expected incidence of 9% in neonates with HIE [4,5,6,7]. A sample size of 138 was obtained, assuming a statistical power of 80% and an alpha value of 0.05. Categorical data were reported as absolute numbers with corresponding percentages. Continuous data were reported as mean and standard deviation or as median and interquartile range according to distribution, analyzed using the Shapiro–Wilk test. Categorical data were compared using the chi-square test or Fisher’s exact test, as appropriate; continuous data were compared using the independent Student’s *t*-test or the Mann–Whitney test, based on the result of the Shapiro–Wilk test. For each significant risk factor, univariate logistic regression analysis was performed to calculate the odds ratio (OR) and the corresponding 95% confidence intervals (CIs) to quantify the association between the risk factor and SNHL. Finally, to adjust for confounding variables, Firth’s penalized logistic regression analysis was performed to identify independent predictive factors for SNHL. Variables were selected a priori based on clinical relevance and previously reported associations with hearing loss. Given the limited number of outcome events, the number of variables included in the model was intentionally restricted. A *p*-value of <0.05 was considered statistically significant. Statistical analysis was performed using the Statistical Package for Social Science (SPSS^®^, IBM^®^) version 25.

## 3. Results

During the study period, 164 newborn infants met the inclusion criteria. Fifteen infants were excluded: 7 due to missing relevant study data, 3 who died before audiological screening, and 5 whose parents denied permission for anonymous data use. One hundred forty-nine infants were included, and their data were analyzed. Among these 149 infants, 6 were diagnosed with hearing loss (HL group) at three months of age, while 143 had normal hearing (normal hearing group, NH group).

The incidence of hearing loss in HIE infants at our center was 4.0% (6/149; 95% CI 1.5–8.4%, calculated using the Clopper–Pearson exact method). Previously published studies report incidences ranging from 8.8% to 19%. Considering the combined data from these studies (57/409), the overall reported incidence is approximately 13.9% (95% CI 10.7–17.6%). A chi-square comparison indicated a lower incidence in our cohort (χ^2^ = 10.69, df = 1, *p* = 0.001), although this comparison should be interpreted with caution due to differences in study design, definitions of hearing loss, and follow-up duration.

Demographic and neurological screening data for the entire population are shown in Table 1.

Caesarean delivery occurred in 38.3% of cases; maternal disease was present in just over half of pregnancies (55.0%), and perinatal asphyxia sentinel events were present in 41.6% of cases. Moderately abnormal aEEG was present in most cases (79.9%), while severely abnormal aEEG and burst suppression anomaly were present in 16.1% and 4.0% of cases, respectively. The overall incidence of HL at audiological ABR at three months of age was 4.0%.

Comparing the HL and NH groups (Table 2), newborns with HL had a lower gestational age than NH newborns, although this difference was not statistically significant. Caesarean sections occurred significantly more frequently in newborns with HL.

The HL group also had significantly lower Apgar scores at 1, 5, and 10 min compared to the NH group and more frequently exhibited burst suppression anomalies on aEEG monitoring.

Clinical data and therapies for our patients appear in Table 3.

Infants with HL were more frequently affected by multi-organ involvement than normal hearing infants, as indicated by higher rates of RDS, pulmonary hypertension, cholestasis, hypotension requiring inotropic support, anemia, thrombocytopenia and coagulopathy requiring transfusions, and hyperglycemia requiring insulin. Regarding respiratory aspects, 39 out of 149 babies (26.2%) received invasive mechanical ventilation, with significantly more neonates with HL (5/6; 83.3%) compared to 34 out of 143 (23.8%). Additionally, infants with HL differed significantly from the NH group, as they received furosemide more frequently and required a longer duration of oxygen therapy. Infants with HL achieved full enteral feeding later and had a longer duration of parenteral nutrition and hospital stay. Short-term neurological findings are shown in Table 4.

Infants with HL had a higher incidence of seizures, indicating more severe neurological involvement. Just over one-third of infants with HL had abnormal PLIC, while 50% had lesions predictive of abnormal neurodevelopmental outcomes.

Univariate logistic regression analysis showed that several risk factors were associated with HL. However, gestational age, 5 min Apgar score, and lesions predictive of abnormal outcome were associated with the odds of HL in Firth’s penalized logistic regression analysis (Table 5). The overall Firth’s penalized logistic regression model was statistically significant (*p* < 0.001). However, these associations may also reflect the overall severity of the disease rather than specific causal mechanisms, given that even penalized statistical models have limitations in cases with a low number of events.

Regarding audiological follow-up (Table S1), among six cases with hearing loss at three months of age, one infant with moderate unilateral hearing loss improved to normal hearing by nine months, while another progressed from mild-moderate bilateral hearing loss to moderate unilateral hearing loss within seven months, with stable results at subsequent assessments. These cases could be interpreted as transient synaptic/neural dysfunction (Patient 1) and bilateral sensory dysfunction (Patient 2). Based on clinical monitoring and parental opinion, speech therapy was initiated for both infants.

The remaining four infants diagnosed with permanent bilateral sensorineural hearing loss (SNHL) (three moderate, one severe) were fitted with hearing aids at 5 ± 1.4 months of age. The prevalence of bilateral SNHL at six months was therefore 2.7% (4/149) (Table S1). In the normal hearing group at three months of age, none of the 143 infants showed permanent hearing loss at the end of audiological follow-up, and only seven cases of mild to moderate transient conductive hearing loss (three bilateral, four unilateral) were observed.

In our study, we did not find any cases of permanent auditory neuropathy spectrum disorder or delayed hearing loss during the study period. Regarding the latter, all infants who received a “pass” result on the pre-discharge TEOAEs (125 of 149; 83.8%) were confirmed to have normal hearing at the end of follow-up.

Among the 24 infants (16.1%) who received a “refer” result on the pre-discharge TEOAEs, 19 (79%) showed normal hearing at follow-up evaluation. The remaining 5 cases with an initial “refer” result were confirmed, as previously described, to have permanent sensorineural hearing loss (bilateral in 4 infants, unilateral in 1 infant).

## 4. Discussion

HIE is considered a severe condition associated with significant morbidity and mortality. Hypoxia is known to cause irreversible cellular damage to the cochlea, although there is no clear indicator for the development of this damage [24,25].

Our data suggest that moderate and severe HIE may be risk factors for auditory disorders compared to the general population [26].

We found a “Refer” rate of 16.1% (24/149) at the first audiological evaluation (TEOAEs). This incidence was similar to the 19% reported by Simsek et al. [6] who also distinguished incidence among Sarnat & Sarnat stage 1 (19.6%), stage 2 (19%), and stage 3 (23.8%) groups. The percentage we found is considerably higher than the “gold standard” proposed by JCIH and the rate previously observed in our experience with healthy term infants [27,28].

Through accurate audiological follow-up, we found a 4% (6/149) prevalence of hearing loss at three months of age. Bilateral sensorineural hearing loss requiring hearing aids was later confirmed in four infants (2.7%), while in one case the diagnosis of unilateral sensorineural hearing loss was confirmed, and in the last patient, hearing normalized in both ears. It is important to emphasize that, as these infants are at high risk of developing auditory neuropathy, audiological follow-up should always include ABR assessment.

Our study found a lower incidence of hearing impairment among HIE infants than previously reported [5,6,7,8,9].

This may be due, at least in part, to the standardization of care for newborns with HIE, as the Fondazione Policlinico A. Gemelli IRCCS in Rome is a historic regional referral center for TH. This has enabled us to personalize all treatments and procedures. Specifically, we provided noninvasive respiratory support as a first-line option to all newborns with respiratory failure, reserving mechanical ventilation for only the most severe cases [29].

The comparison with previously published studies should be interpreted with caution. Differences in study design, definitions of hearing loss, screening methods, and follow-up duration may introduce heterogeneity. Therefore, the aggregated incidence from these studies serves as an approximate reference rather than a formal pooled estimate from a systematic review or meta-analysis. Moreover, we did not find any cases of delayed hearing loss within the first year of life, and all infants with permanent hearing loss had a “Refer” result in the previous pre-discharge screening. This result differs from that of Michniewicz et al. [30], who found some normal pre-discharge TEOAE results after TH, followed by hearing impairment during audiological follow-up, indicating delayed or progressive damage due to HIE. We found that 79% (19/24) of infants classified as “Refer” at pre-discharge screening became “Pass” at subsequent evaluation, suggesting transient hearing damage or developmental changes in the auditory system, as already observed in preterm infants [31,32].

According to previous data [8,33,34], we found that our infants with hearing impairment had a more severe degree of asphyxia, defined as a lower APGAR score not only at 10 min, but also at 1 and 5 min of life. Our patients with HL also had a higher incidence of severe HIE. Caesarean section was more common in newborns with HL; however, this finding does not appear to be clinically significant and is likely due to the small sample size.

In addition, we found that the aEEG tracing was more frequently abnormal in infants with HL, suggesting an association between audiological risk and severely compromised aEEG, similar to Ouwehand et al. [35] who reported the predictive value of aEEG for long-term neurological outcome.

In our study, infants with HL had a more severe post-asphyxial syndrome and a worse clinical course, as indicated by pulmonary hypertension, coagulation changes, need for mechanical ventilation, and longer hospital stays. In contrast, antibiotic therapy does not significantly distinguish HL infants from normal infants. This result can likely be interpreted as a consequence of the protective dosage and short duration of antibiotic therapy used in our practice.

Loop diuretics can damage the cochlea, and their use is commonly considered an audiological risk factor [24]. We found that furosemide administration was significantly higher in our infants with hearing loss. However, our infants with hearing impairment had a more complicated clinical course, particularly regarding respiratory function, and the need for diuretic treatment could be considered a proxy for severe multi-organ damage rather than an audiological risk factor in itself.

The frequency of auditory or visual disturbances was previously found to be higher in hyperglycemic and hypoglycemic HIE infants [36]. In our population, there were fewer infants with hypoglycemia (22.8%) compared with other studies [37], and the incidence was not higher in infants with HL than in those with normal hearing. However, infants with sensorineural HL had a higher incidence of hyperglycemia requiring insulin therapy compared with infants with normal hearing. This could also be interpreted as a consequence of the blood glucose response to the stress of a more severe post-asphyxia syndrome. Chakkarapani et al. [38] suggested that hypoglycemia, hyperglycemia, and glycemic lability are common in infants who undergo TH for HIE. It was also reported that hyperglycemia may be more harmful than hypoglycemia in terms of brain damage and may be associated with greater damage to the basal ganglia, thalami, and posterior limb of the internal capsule, rather than the white matter or cortex.

Historically, brain damage following HIE was described with two distinct patterns on MRI evaluation. The first, called the central pattern, results from an abrupt reduction in cerebral blood flow associated with greater severity of HIE and involves the BGT, PLIC, perirolandic cortex, and hippocampus. The second, called the parasagittal pattern, results from a more moderate decrease in cerebral blood flow associated with milder HIE and involves the watershed white matter and gray matter. Rutherford and colleagues developed an MRI score, used in our study, showing that moderate or severe lesions in the BGT, an abnormal PLIC, or severe white matter lesions were predictive of an abnormal neurodevelopmental outcome [21,22,23].

By applying the MRI score proposed by Rutherford et al., we found through logistic regression that the presence of lesions predictive of abnormal neurological outcomes may also be a risk factor for unfavorable audiological outcomes. In particular, infants with SNHL showed greater involvement of the BGT and PLIC, indicating a central pattern likely due to higher severity of HIE. This may serve as a preliminary observation for future neuroimaging studies.

Furthermore, a shorter time to full enteral feeding and parenteral nutrition in infants with normal hearing could indicate a beneficial effect of minimal enteral feeding during hypothermia on auditory outcomes, as previously suggested [39,40].

Multiple logistic regression analysis ultimately showed that lower gestational age influenced the association between hearing impairment and HIE treated with therapeutic hypothermia, and that the incidence of hearing loss increases as gestational age decreases. This finding is biologically plausible, as more immature auditory structures at lower gestational ages may be more susceptible to pathological agents, as has already been suggested for preterm and very low birth weight infants [41]. Nevertheless, this result should be interpreted with caution due to the limited range of gestational ages and the low incidence of HL.

Our study has several strengths and some limitations that should be considered. The main limitation is its retrospective, single-center design. Additionally, infants with hearing disorders did not undergo genetic testing, despite having no suggestive family history, so it cannot be certain that all cases of HL were attributable to HIE. As 11 newborns were excluded due to lack of consent or missing data, potential selection bias cannot be ruled out. Similarly, selection bias cannot be excluded when considering the three infants who died, as they likely had a more severe clinical presentation and were therefore at higher risk of hearing impairment. Furthermore, audiological follow-up did not continue beyond the age of one year. Given the low incidence of HL, the reliability of the univariate and multivariate statistical analyses is limited and should be confirmed in a larger study population. However, our study also has several strengths. The sample size was appropriate. Data were obtained from a homogeneous population with similar neonatal care and treatments and were collected from medical records rather than interviews or questionnaires. Audiological data were obtained through accurate diagnostic evaluation and thorough, long-term follow-up.

## 5. Conclusions

Infants with HIE are at increased risk for hearing disorders, which are related to the severity of the condition. These patients require careful neonatological and audiological management. Audiological assessment should be accurate in both methods and timing to avoid overestimating the prevalence and degree of HL, particularly regarding therapeutic and rehabilitative decisions. Personalized management could address specific neonatal risk factors. The association between gestational age and susceptibility to cochlear damage should be confirmed by further studies.

## Figures and Tables

**Table 1:** Demographic data and neurological screening of the study population.

	N 149
Gestational age, weeks	39.0 ± 1.6
Birth weight, grams	3283 ± 520
Male	94 (63.1%)
Cesarean section	57 (38.3)
Small For Gestational Age	19 (12.8%)
Large For Gestational Age, n	16 (10.7%)
1 min Apgar score	4 [2–6]
5 min Apgar score	7 [5–8]
10 min Apgar score	7 [6–8]
Arterial Cord pH	6.9 ± 0.6
Arterial Cord Base Deficit, mmol/L	15 ± 6
Maternal metabolic disease	33 (22.1%)
Maternal infectious disease	38 (25.5%)
Maternal cardiovascular disease	11 (7.4%)
Perinatal asphyxia sentinel events	62 (41.6%)
Moderate HIE	134 (89.9%)
Severe HIE	15 (10.1%)
Moderately abnormal aEEG	119 (79.9)
Severely abnormal aEEG	24 (16.1%)
Burst Suppression aEEG	6 (4%)
TEOAE screening failure at discharge	24 (16.1)
HL at 3 months of life	6 (4.0)

Values are expressed as mean and standard deviation or as median [interquartile range] or number (%). HIE: Hypoxic-ischaemic encephalopathy; aEEG: amplitude-integrated electroencephalogram; TEOAE: transient evoked oto-acoustic emissions; HL: hearing loss.

**Table 2:** Demographic data and neurological screening of infants with and without HL.

	HL Group N 6	NH Group N 143	*p* Value
Gestational Age, weeks	37.5 [36.5–38.8]	39.0 [38.0–40.0]	0.052
Birth weight, grams	2775 [2515–3750]	3300 [2940–3570]	0.243
Male	5 (83.3%)	89 (62.2%)	0.414
Cesarean section	6 (100)	51 (35.7)	*0.003*
Small For Gestational Age	1 (16.7%)	18 (12.6%)	0.566
Large For Gestational Age	1 (16.7%)	15 (10.5%)	0.500
Maternal metabolic disease	1 (16.7%)	32 (22.4%)	1
Maternal infectious disease	3 (50%)	35 (23.8%)	0.103
Maternal cardiovascular disease	1 (16.7%)	10 (7.0%)	0.328
Perinatal asphyxia sentinel events	5 (83.3%)	57 (39.9%)	0.083
1 min Apgar score	2 [1–4]	4 [2–6]	*0.027*
5 min Apgar score	4 [1–6]	7 [5–8]	*0.010*
10 min Apgar score	7 [3–7]	7 [6–8]	*0.035*
Arterial Cord pH	6.95 [6.62–7.16]	7.06 [6.93–7.14]	0.298
Arterial Cord Base Deficit, mmol/L	15.5 [13.7–25.1]	15.0 [12.4–18.7]	0.402
Moderate HIE	4 (66.7%)	130 (90.9%)	0.112
Severe HIE	2 (33.3%)	13 (9.1%)	0.112
Moderately abnormal aEEG	3 (50.0)	116 (81.1)	0.065
Severely abnormal aEEG	1 (16.7)	23 (16.1)	*1*
Burst Suppression aEEG	2 (33.3)	4 (2.8%)	*0.019*
Respiratory rate SD	10.3 ± 8.8	10.4 ± 7.1	0.915

Values are expressed as median [interquartile range] or number (%). Data were compared using the Mann–Whitney test or Fisher’s exact test. HIE: Hypoxic-ischaemic encephalopathy; aEEG: amplitude-integrated electroencephalogram.

**Table 3:** Clinical data and therapies of infants with and without HL.

	HL Group N 6	NH Group N 143	*p* Value
Respiratory distress syndrome	3 (50%)	14 (9.8%)	*0.025*
Pulmonary hypertension	3 (50%)	7 (4.9%)	*0.004*
Pulmonary air leak	1 (16.7%)	4 (2.8%)	0.189
Mechanical ventilation	5 (83.3%)	34 (23.8%)	*0.005*
Oxygen therapy duration	12 [2–55]	0 [0–0]	*<0.001*
Systemic hypotension needing inotropic support	4 (66.7%)	7 (4.9%)	*<0.001*
Anemia needing RBC transfusion	3 (50%)	14 (9.8%)	*0.020*
Thrombocytopenia needing PLTs transfusion	4 (66.7%)	8 (5.6%)	*<0.001*
Coagulopathy needing FFP transfusion	5 (83.3%)	17 (11.9%)	*<0.001*
Hypoglycemia	2 (33.3%)	32 (22.4%)	0.620
Hyperglycemia needing insulin	2 (33.3%)	5 (3.5%)	*0.026*
Cholestasis	2 (33.3%)	0	*0.001*
Proven early onset sepsis	0	7 (4.9)	*1*
Antibiotic therapy	6 (100)	143 (100)	*1*
Cycles of antibiotic therapy per infant	2 [1–2]	2 [1–2]	*0.306*
Acute renal failure	1 (16.7%)	3 (2.1%)	0.154
Diuretic therapy	2 (33.3%)	4 (2.8%)	*0.019*
Parenteral nutrition duration, days	9 [5–16]	5 [4–5]	*0.015*
Full enteral feeding, days	12 [11–18]	7 [6–8]	*<0.001*
Length of hospital stay, days	26 [16–66]	12 [10–15]	*0.001*

Values are expressed as median [interquartile range] or number (%). Data were compared using the Mann–Whitney test or Fisher’s exact test. RBC: red blood cells; PLTs: platelets; FFP: fresh frozen plasma.

**Table 4:** Short-term neurological outcomes.

	HL Group N 6	NH Group N 143	*p* Value
Seizures	5 (83.3%)	40 (28.0%)	*0.010*
Day of life on which MRI was performed	11 [8–20]	9 [8–11]	0.415
Basal ganglia-thalamus normal	2 (33.3)	125 (87.4)	*0.005*
Basal ganglia-thalamus mild	2 (33.3)	10 (7.0)	*0.075*
Basal ganglia-thalamus moderate	1 (16.7)	7 (4.9)	*0.286*
Basal ganglia-thalamus severe	1 (16.7)	1 (0.7)	*0.079*
PLIC normal	3 (50.0)	125 (87.4)	*0.037*
PLIC equivocal	1 (16.7)	14 (9.8)	*0.477*
PLIC abnormal	2 (33.3)	4 (2.8)	*0.040*
White matter normal	4 (66.7)	106 (74.1)	*0.651*
White matter mild	0	11 (7.7)	*1*
White matter moderate	1 (16.7)	22 (15.4)	*1*
White matter severe	1 (16.7)	4 (2.8)	*0.188*
Cortex normal	5 (83.3)	125 (87.4)	*1*
Cortex mild	0	5 (3.5)	*1*
Cortex moderate	1 (16.7)	6 (4.2)	*0.255*
Cortex severe	0	7 (4.9)	*1*
Hemorrhage	2 (33.3%)	69 (48.3%)	0.683
Lesions predictive of abnormal outcome	3 (50%)	11 (7.7%)	*0.011*

Values are expressed as median [interquartile range] or number (%). Data were compared using the Mann–Whitney test or Fisher’s exact test. MRI: magnetic resonance imaging. PLIC: posterior limb of the internal capsule.

**Table 5:** Logistic regression analysis.

**Univariate Logistic Regression Analysis of Risk Factors for HL**	**Odds Ratio**	**95% CI**	***p***
	17.3	2.4–123.3	*0.005*
Apgar score 5 min < 3	16.8	2.9–96.6	*0.002*
Mechanical ventilation	16.0	1.8–141.9	*0.013*
Pulmonary hypertension	19.4	3.3–114.2	*<0.001*
Systemic hypotension	38.9	6.1–249.5	*<0.001*
Anemia	9.2	1.7–50.1	*0.010*
Thrombocytopenia	33.8	5.4–212.7	*<0.001*
Coagulopathy	37.1	4.1–336.5	*0.001*
Diuretic therapy	17.4	2.4–124.2	*0.029*
Hyperglycemia needing insulin	13.7	2.0–93-2	*0.007*
Seizures	12.9	1.5–113.7	*0.021*
Lesions predictive of abnormal outcome	12.0	2.16–66.6	*0.005*
Multiple Logistic Regression Analysis for HL after Adjusting for Confounders *	**Odds Ratio**	**95% CI**	***p***
Gestational age	0.49	0.22–0.91	*0.023*
5 min Apgar score	0.52	0.32–0.77	*0.001*
Lesions predictive of abnormal outcome	8.6	1.37–62	*0.023*

* Firth’s penalized logistic regression analysis.

## Data Availability

All data generated or analysed during this study are included in this published article and its Supplementary Materials files.

## Supplementary Materials

The following supporting information can be downloaded at https://www.mdpi.com/article/10.3390/jcm15093180/s1: Table S1. Main audiological data and follow-up of the six patients with hearing loss.
