# Supplementary material for: Hypoxic Ischemic Encephalopathy: Hearing Impairment and Related Risk Factors
Source: J Clin Med. 2026 Apr 22;15(9):3180. doi: 10.3390/jcm15093180 (PMC13164453; doi:10.3390/jcm15093180)
Supplement: Supplementary file 1 [file jcm-15-03180-s001.zip › jcm-4185597-supplementary.pdf]

Table S1. Main audiological data and follow-up of the six patients with hearing loss

		3-4 months	6-7 months	9-10 months	12-13 months
**Patient 1**	**TEOAE**	normal		normal	normal
	**ABR**	UHL<br>left ear 50 dB	-	normal	normal
**Patient 2**	**TEOAE**	left ear refer<br>right ear refer	left ear normal<br>right ear refer		left ear normal<br>right ear refer
	**ABR**	HL<br>left ear (40 dB)<br>right ear (60 dB)	UHL<br>right ear (60 dB)	-	UHL<br>right ear (60 dB)
**Patient 3**	**TEOAE**	left ear refer<br>right ear refer	left ear refer<br>right ear refer		left ear refer<br>right ear refer
	**ABR**	SNHL<br>left ear (60 dB)<br>right ear (70 dB)	SNHL<br>left ear (60 dB)<br>right ear (70 dB)		SNHL<br>left ear (60 dB)<br>right ear (70 dB)
**Patient 4**	**TEOAE**	left ear refer<br>right ear refer	left ear refer<br>right ear refer		left ear refer<br>right ear refer
	**ABR**	SNHL<br>left ear (60 dB)<br>right ear (60 dB)	SNHL<br>left ear (60 dB)<br>right ear (60 dB)		SNHL<br>left ear (60 dB)<br>right ear (60 dB)
**Patient 5**	**TEOAE**	left ear refer<br>right ear refer	left ear refer<br>right ear refer		left ear refer<br>right ear refer
	**ABR**	SNHL<br>left ear (60 dB)<br>right ear (60 dB)	SNHL<br>left ear (60 dB)<br>right ear (60 dB)		SNHL<br>left ear (60 dB)<br>right ear (60 dB)
**Patient 6**	**TEOAE**	left ear refer<br>right ear refer	left ear refer<br>right ear refer		left ear refer<br>right ear refer
	**ABR**	SNHL<br>left ear (80 dB)<br>right ear (80 dB)	SNHL<br>left ear (80 dB)<br>right ear (80 dB)		SNHL<br>left ear (80 dB)<br>right ear (80 dB)

HL: hearing loss. UHL: unilateral hearing loss; SNHL: bilateral sensorineural hearing loss
